# Zebrafish promoter microarrays identify actively transcribed embryonic genes

**DOI:** 10.1186/gb-2006-7-8-r71

**Published:** 2006-08-04

**Authors:** Fiona C Wardle, Duncan T Odom, George W Bell, Bingbing Yuan, Timothy W Danford, Elizabeth L Wiellette, Elizabeth Herbolsheimer, Hazel L Sive, Richard A Young, James C Smith

**Affiliations:** 1 Wellcome Trust/Cancer Research UK Gurdon Institute and Department of Zoology, Cambridge University, Cambridge CB2 1QN, UK; 2 Whitehead Institute for Biomedical Research, Nine Cambridge Center, Cambridge, MA 02142, USA; 3 Computer Science and Artificial Intelligence Laboratory, Massachusetts Institute of Technology, Vassar Street, Cambridge, MA 02139, USA; 4 Novartis Institutes for Biomedical Research, Mass Ave, Cambridge, MA 02139, USA

## Abstract

We have designed a zebrafish genomic microarray to identify DNA-protein interactions in the proximal promoter regions of over 11,000 zebrafish genes. Using these microarrays, together with chromatin immunoprecipitation with an antibody directed against tri-methylated lysine 4 of Histone H3, we demonstrate the feasibility of this method in zebrafish. This approach will allow investigators to determine the genomic binding locations of DNA interacting proteins during development and expedite the assembly of the genetic networks that regulate embryogenesis.

## Abstract 2

The development and verification of a genomic microarray for ChIP-chip analysis of zebrafish genes is described.

## Background

As the development of an organism proceeds from the fertilized egg to multicellular embryo, cascades of gene activation, triggered in response to localized determinants and extracellular signals, lead to changes in gene expression in groups of cells. These changes in gene expression eventually direct the course of cell differentiation [1]. Gene regulatory networks (GRNs), which detail the inputs into the cis-regulatory sites of each gene in a particular cell type at a particular time during development, are increasingly being used to describe the process of development and to provide a basis for testing models of gene expression [1]. For instance, GRNs have recently been created to describe mesendoderm formation in sea urchin and *Xenopus *embryos [2-4], segmentation in *Drosophila *and vulval development in *Caenorhabditis elegans *(reviewed in [5]). These networks have been built using a combination of knock-down and over-expression analyses, expression arrays, promoter analyses, bioinformatics and some direct promoter binding data. However, detailed knowledge of the direct binding of developmental regulatory proteins at promoters and enhancers in the genome is very limited at present. Having such knowledge, linked to functional gene expression data, will increase our ability to test predictions made by network models of embryonic development and to refine further our understanding of this complex process [6].

One approach to identify genomic regions bound by transcription factors and other DNA binding proteins is chromatin immunoprecipitation (ChIP), which, when combined with genomic microarrays, provides extensive information on genomic binding and allows identification of active or repressed genes and the elucidation of transcriptional regulatory networks. This approach, known as ChIP-chip or genome-wide location analysis, has been widely used in yeast, *Drosophila *and mammalian cells to study gene regulation, histone modification and localized binding of specific transcription factors as cells differentiate or respond to environmental signals (for example, [7-16]). Here we demonstrate the application of this powerful, genome-wide approach in an equally powerful model system, the zebrafish.

Zebrafish are firmly established as an important and informative model system for studying vertebrate embryogenesis and organogenesis, as well as modeling human disease (for example, [17-21]). Among the advantages of zebrafish are the ease with which large numbers of embryos can be obtained and the *ex utero *development of the embryos. Together these allow manipulation at stages when many other vertebrate models, such as the mouse, are inaccessible. In addition, large-scale mutagenesis screens have generated many mutants in embryonic development [22-25], and expressed sequence tag (EST) projects and sequencing of the genome have brought zebrafish into a post-genomic era that can now be exploited. Finally, the ability to generate, inexpensively, large numbers of transgenic embryos carrying promoter reporter constructs make zebrafish an ideal model system for functional studies of transcriptional regulation networks [26]. For instance, zebrafish can be used to make transgenic animals, both as transient and stable lines, to study reporter gene expression under the control of regulatory sequences.

Here we describe the design of a genomic microarray representing a substantial fraction of zebrafish promoter regions and we go on to verify this microarray using an antibody directed against a trimethylated form of Histone H3. Covalent modification of histone tails causes alterations in the structure of chromatin, which in turn regulate the availability of regions of DNA to specific and general transcription factors. One example is the trimethylation of lysine 4 in the tail of Histone H3 (H3K4Me3), which serves as a binding site for the SAGA and SLIK histone acetyltransferase complexes and Iswi chromatin remodeling ATPase in yeast [27,28]. This chromatin mark is associated with actively transcribed genes in both yeast and higher eukaryotes [9,10,29], and genome-wide binding data and detailed studies of individual gene loci have shown that H3K4Me3 is specifically localized to the 5' end of transcribed genes in eukaryotes [9,30,31].

To verify the arrays and to show the utility of this microarray resource in zebrafish, we used ChIP directed against tri-methylated K4 Histone H3. Since the gastrula stage embryo expresses thousands of genes [32,33], using H3K4Me3 allows us both to confirm the usefulness of the technique and our microarray design and to identify those genes that are potentially actively transcribed in the embryo. We show that 4,735 genes of the 11,117 represented on our microarray are marked by H3K4Me3 in gastrula stage embryos, suggesting that these genes are expressed. This approach not only identifies genes that are expressed ubiquitously and/or at high levels, but also allows us to identify genes that are expressed in a subset of the cells of the embryo.

This paper is the first to describe chromatin immunoprecipitation combined with genomic microarrays in zebrafish and the use of an antibody against tri-methylated K4 Histone H3 validates the technique and resource for future use. In particular we hope that this approach can be applied to specific transcription factors and many other chromatin marks or DNA binding proteins during zebrafish development.

## Results and discussion

### Optimization of chromatin immunoprecipitation in zebrafish embryos

Before testing our genomic microarrays, it was first necessary to optimize a ChIP protocol and assess the effectiveness of conventional ChIP in zebrafish embryos. For this we used gastrula stage zebrafish and a ChIP protocol [34] that we modified for zebrafish with a well-characterized antibody directed against H3K4Me3 (see Materials and methods for further information), a marker of the 5' end of actively transcribed genes. We then performed PCR analysis on the purified DNA using primers for the promoter region of genes known to be expressed or not expressed during gastrulation. The results show that we could reliably detect expressed genes, such as *bactin2 *and *wnt11*, and that non-expressed genes such as *rhodopsin *lacked the H3K4Me3 histone mark (Additional data file 1). During the course of these experiments we also performed control ChIP experiments with an anti-HA antibody and with normal rabbit serum and saw no significant enrichment of expressed genes (not shown).

For larger scale ChIP for microarray experiments, we used 1,000 embryos per sample for anti-histone immunoprecipitation. Previous reports of ChIP combined with microarrays have used approximately 1 × 10^7 ^to 5 × 10^8 ^cells for each ChIP [10,12,14,15]. Because the number of cell divisions between the start and end of gastrulation in zebrafish is known, we can estimate that a mid-late gastrula stage embryo contains approximately 8,000 to 16,000 cells, and our anti-histone experiments, therefore, used approximately 8 × 10^6 ^to 1.6 × 10^7 ^cells in each ChIP-chip assay.

### Design of genomic microarrays

**Figure 1:**
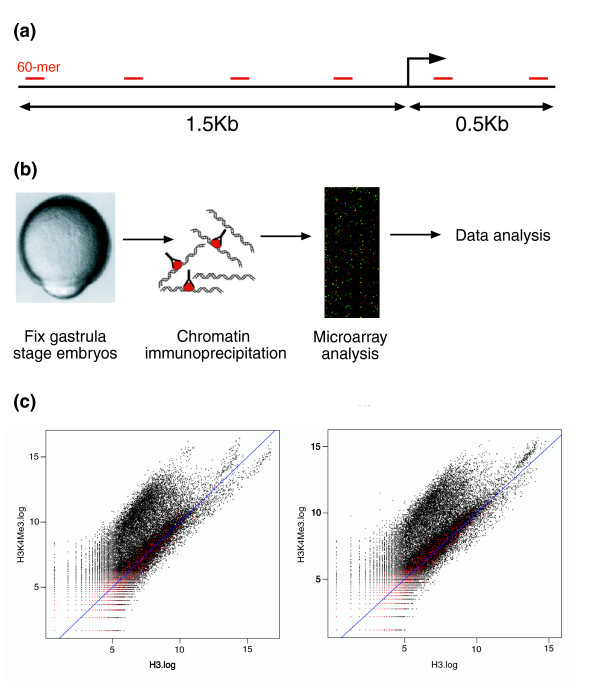
ChIP-chip method in zebrafish embryos. **(a) **Design of promoter arrays: 60-mer oligonucleotides were designed against genomic sequence 1.5 kb upstream and 0.5 kb downstream of the annotated transcription start site of approximately 11,000 zebrafish genes. The resulting probes are arrayed onto two microarray slides. **(b) **ChIP-array protocol. **(c) **Examples of scatter plots obtained from one hybridization of immunoprecipitated DNA on one 2-slide proximal promoter microarray set. Log_2 _ratios for each labeled sample are plotted against each other. Enriched probes are seen above the diagonal. Control spots (zebrafish gene desert and *Arabidopsis *gene probes), shown in red, fall along the diagonal.

The design of our genomic microarrays is described in more detail in Materials and methods. Briefly, 13,413 genes were selected from 5 databases of zebrafish cDNA. These transcripts were mapped to the zebrafish genome (Zv4; July 2004), and the 5' end of each mapped transcript was defined as the transcription start site (TSS). We designed 60-mer probes to represent the region from 1.5 kb upstream to 0.5 kb downstream of the TSS and spaced at approximately 250 base-pair (bp) intervals (Figure 1a). In practice, spacings varied because promoters were masked for repetitive sequence, and oligo selection was optimized for parameters such as GC content. A minimum representation of two probes was required for a promoter region to be included in the final design. The final design represents 11,117 promoter regions that, due to redundancy in the genome assembly, map to 12,545 locations across the genome.

The arrays also contain negative control probes designed against gene desert regions, defined as regions of the zebrafish genome most distant from any annotated genes. Additional negative controls were designed to represent *Arabidopsis *genes that show no similarity to zebrafish genes. Finally, the arrays include seven positive control genes with probes printed two to four times on each slide for comparison within and across slides.

In designing the promoter microarrays we selected databases that are considered to contain full-length cDNAs, in order to be confident that the upstream promoter regions were correctly assigned as far as possible. Despite this, because information on the 5' ends of many zebrafish genes is currently incomplete, it is inevitable that this approach will identify some proportion of TSSs incorrectly. However, since zebrafish sequencing projects are still underway, use of probes of known sequence allows remapping as new genome builds are released and mis-targeted promoters can be identified. As sequencing projects are completed and annotation becomes more comprehensive, these arrays can readily be updated to include additional probes or to remove incorrect probes.

### ChIP-chip with anti-H3K4Me3

To establish that these zebrafish arrays could be used to identify regions interacting with DNA binding proteins we performed ChIP with anti-H3K4Me3 (Figure 1b,c). As an input sample with which to compare H3K4Me3 we also performed ChIP with an antibody against Histone H3. This gives a comparison with total nucleosome occupancy across the genome and is a more accurate way to normalize data obtained from histone ChIPs [9,35].

Our study identified 4,735 genomic regions occupied by H3K4Me3 and, therefore, potentially active at gastrula stages (Additional data file 5). On the one hand, this will slightly under-estimate the number of genes associated with H3K4Me3 since, in some cases, one 'bound region' might be associated with two gene promoters on opposite strands. On the other hand, since this list of bound regions is partially redundant due to some duplication of regions in the Zv4 genome assembly (see above), 4,735 is likely to over-estimate the actual number of genes bound by H3K4Me3. This figure is, however, consistent with the previous analysis by Mathavan and colleagues [33] of the number of zebrafish transcripts during gastrulation. These authors found that 3,035 genes represented on their expression arrays were zygotically expressed during development. Of our 4735 genes, 1,070 are also identified by transcriptome analysis [33]. This difference is likely to be due in large part to the different sets of genes represented on our arrays, which is a consequence of different design strategies; of the 3,035 zygotically expressed genes that were identified by Mathavan and colleagues, 1,224 are represented on our array. This suggests that we failed to identify approximately 13% of those genes identified by transcriptome analysis; this may be due to calling false negatives (see analysis below) or because some of those genes identified as zygotically active are expressed after gastrulation.

### Validation of microarray data and estimation of false positive and false negative rates

**Figure 2:**
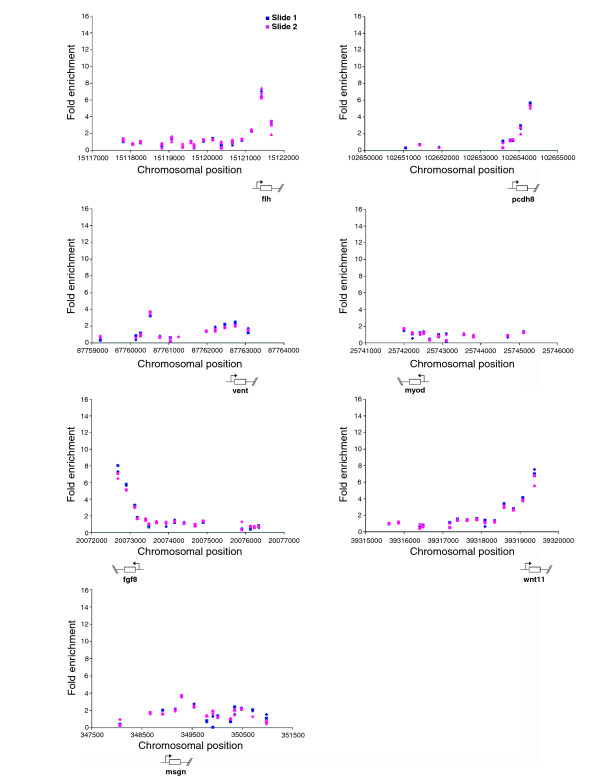
Positive control replicates show similar enrichment values. For positive control genomic regions each point shows unprocessed ChIP-enrichment ratios for probes on each slide (weighted average across three replicates [58]). The chromosomal position (based on Zv4 genome assembly annotation) is shown below each graph. The x-axes are not to scale.

Each microarray contained probes designed around the TSS of seven positive control genes, with each probe being spotted between two and four times on each microarray. These control genes (*wnt11*, *vent*, *fgf8*, *flh*, *myod*, *msgn *and *pcdh8*) are all expressed at different levels and in different spatial patterns in late gastrula embryos [36-42]. Figure 2 shows that within and across each microarray calibration spots were very similar, showing reliability and reproducibility in the array data.

Of these seven positive controls, six were called as marked by H3K4Me3, with MyoD not being called. However, at late gastrula stages *myod *is expressed in just a small patch of adaxial cells, which may account for the low levels of H3K4Me3 detected [43]. However some false negative, such as *myod*, and false positive calls are inevitable with a high-throughput microarray approach; we therefore sought to quantify their rates in these experiments.

#### Estimation of false negative and false positive rates

It is difficult to estimate false negative and false positive rates since this relies on prior knowledge of all genes that are occupied by H3K4Me3 in the gastrula stage embryo. None-the-less, since H3K4Me3 occupancy correlates with actively transcribed genes [9,31], we reasoned that genes that are not expressed at gastrula stages would in general not have associated H3K4Me3, whilst those that are expressed would in general be associated with this chromatin mark.

We therefore prepared a list of genes that are believed not to be expressed at gastrula stages ('non-expressed controls'; Additional data file 6) and genes that are known to be expressed at gastrula stages ('expressed controls'; Additional data file 7) in order to compare them with the H3K4Me3 binding data (see Materials and methods). These controls were selected from microarray expression data covering the first 48 hours of zebrafish development [33]. Expressed controls were selected from those that showed peak expression at gastrula stages plus an additional list of mesodermal genes expressed at gastrula stage derived from the Zebrafish Information Network website [44]. 'Non-expressed' genes had no significant expression during gastrulation, according to previous analyses.

**Figure 3:**
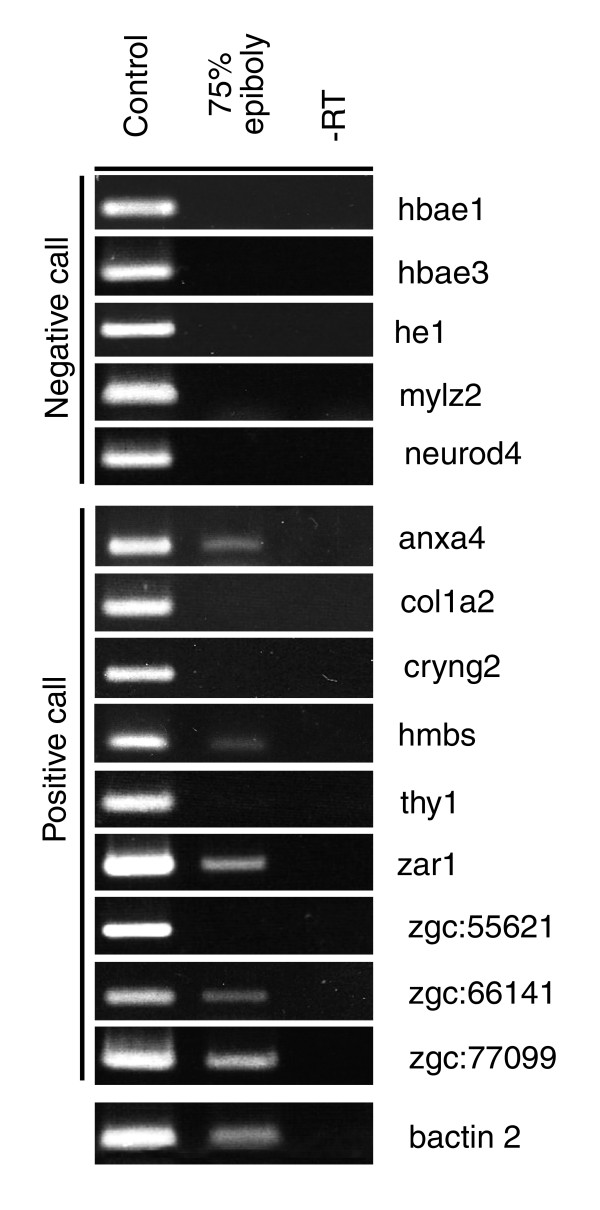
RT-PCR of nine selected 'non-expressed' genes reveals five are expressed at gastrula stages. Real-time RT-PCR analysis was carried out on 75% to 85% epiboly zebrafish embryos.

Of the 86 predicted non-expressed controls, we found that 15 appeared to be tri-methylated at their transcription start site (Additional data file 6). However, we could not rule out the possibility at this point that some of the genes selected as non-expressed controls are in fact expressed at gastrula stages but fell below detection thresholds in other analyses. To test whether any of these genes are indeed expressed at gastrula stages, we performed semi-quantitative RT-PCR analysis on gastrula stage embryos with primers directed against nine of the expected non-expressed genes (*anxa4*, *col1a2*, *cryng2*, *hmbs*, *thy1*, *zar1*, *zgc:55621*, *zgc:661411*, *zgc: 77099*). Five out of the nine non-expressed genes in fact proved to be expressed at gastrula stages to varying degrees (Figure 3), which when extrapolated to all 15 genes leads to an apparent false-positive rate of approximately 9%. This is consistent with previous reports of approximately 5% false positive rates [31,45,46]. We also used primers directed against 5 of the genes called as negative (*hbae1*, *hbae3*, *he1*, *mylz2*, *neurod4*) and *bactin2 *as a positive control gene (which is expressed strongly at gastrula stages). As expected, those genes called as negatives are not expressed, and *bactin2*, a positive control, is expressed at gastrula stages.

One possible explanation for these apparent false positives is that H3K4Me3 binding is not always associated with active gene expression. In embryonic stem cells, for example, some developmentally regulated genes that are associated with H3K4Me3 and H3K4Me2 (another mark of active transcription in eukaryotes) are transcriptionally repressed by an H3K27Me3 mark, as if in a state 'poised' to be transcribed [47,48]. While there is currently no evidence that this also occurs in cells of the developing zebrafish embryo, the possibility that some genes are associated with both activating and repressive marks, and consequently not expressed, is intriguing and currently under investigation.

From the list of expressed controls, 24 out of 123 genes are not marked by H3K4Me3, despite previous evidence from *in situ *and RT-PCR data that these genes are in fact expressed during gastrula stages (Additional data file 7). This suggests a false negative rate of approximately 19%, consistent with previous reports [9,14,15]. There are several reasons why an expressed gene may not be called as marked by H3K4Me3 in this study. For example, these experiments used intact whole gastrula stage embryos that contain approximately 8,000 to 16,000 cells. Genes expressed at low levels or in only a few cells of the embryo, such as *foxa1*, which is expressed at very low levels on the dorsal side of the embryo during gastrulation [49], or *vim*, which can not be detected by *in situ *during gastrulation [32], are unlikely to be identified above background noise. Large-scale expression analysis suffers from the same limitation, since it measures mRNA abundance in a large population of heterogeneous cells, and can, therefore, overlook key changes in a small subset of cells. In addition, some false negatives may derive from incorrect identification of the TSS due to the incomplete annotation of the zebrafish genome, or because alternative start sites of transcription are used in different tissues.

That said, not all genes called as unmarked are expressed at low levels. For example, *cki *and *myf5 *are strongly expressed in the embryo and have good supporting evidence that the most 5' TSS is correctly annotated, but they were not identified in our analysis. However, H3K4Me3 is not inflexibly associated with active genes [9,31,50], and it is possible that some of our false negatives include such genes. In addition, while some genes may be expressed in a subset of cells where transcript levels are high enough to be detected by other analyses, the frequency of H3K4Me3 methylation may be insufficient to be detected by ChIP-chip. Nevertheless, as with all microarray analyses some level of false calls is inevitable. Our error model could be modified to reduce the fraction of false negatives, but we chose to use a stringent threshold to achieve a low false positive rate and to tolerate a higher false negative rate.

### H3K4Me3 marks the 5' end of expressed genes in zebrafish

**Figure 4:**
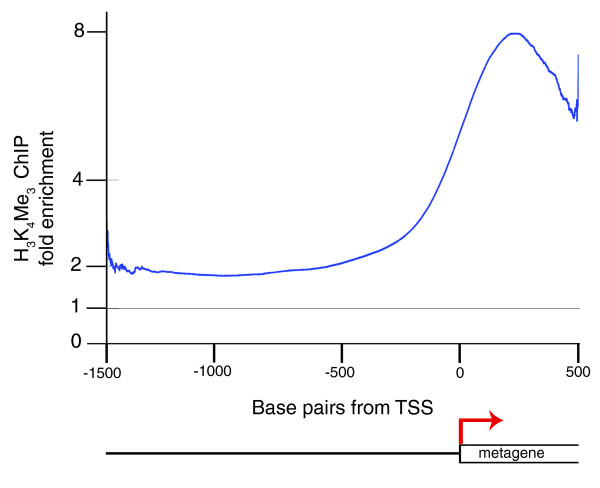
Metagene analysis shows enrichment of H3K4Me3 at the 5' end of the transcribed region. Plot of the averaged signal from all promoter regions occupied by H3K4Me3. This metagene was created from the smoothed and averaged collection of regions identified by the error model as containing H3K4Me3, as described in Materials and methods.

**Figure 5:**
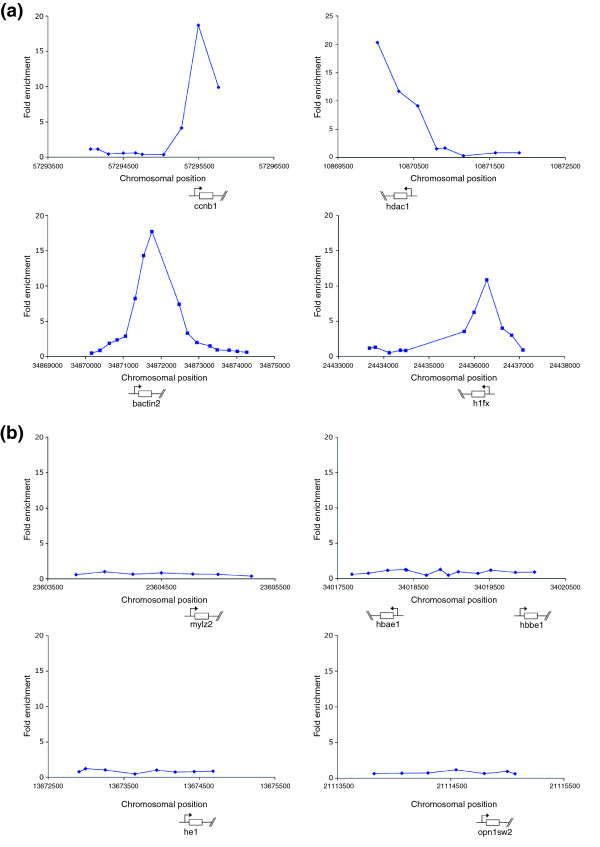
H3K4Me3 is enriched at the 5' end of ubiquitously expressed genes but not enriched at non-expressed genes. **(a) **Examples of genes marked by H3K4Me3 showing a peak of enrichment at the 5'end of the gene. **(b) **Examples of non-expressed genes that are not marked by H3K4Me3. For (a) and (b) plots show unprocessed ChIP-enrichment ratios for all probes within a genomic region. The chromosomal position (based on Zv4 genome assembly annotation) and the transcription start site and direction of transcription for each gene are shown below each graph. The x-axes are not to scale.

In yeast and higher eukaryotes, such as *Drosophila*, chicken and mouse, H3K4Me3 is associated with the 5' end of genes [9,10,30,31]. We confirmed that this is also the case in zebrafish by creating a composite meta-gene from the collection of regions marked by H3K4Me3 (Figure 4). The results show that this chromatin mark occupies regions lying within a few hundred bases of the transcriptional start site. Inspection of individual genes confirms that this enrichment can be found at the 5' end of expressed genes. For instance ubiquitously expressed genes, such as *bactin2 *and *cyclin B1 *(*ccnb1*) are marked by H3K4Me3 at gastrula stages, whereas genes that are not expressed at gastrula stages, such as embryonic globin genes *hbae1 *and *hbbe1*, show no enrichment (Figure 5a,b).

**Figure 6:**
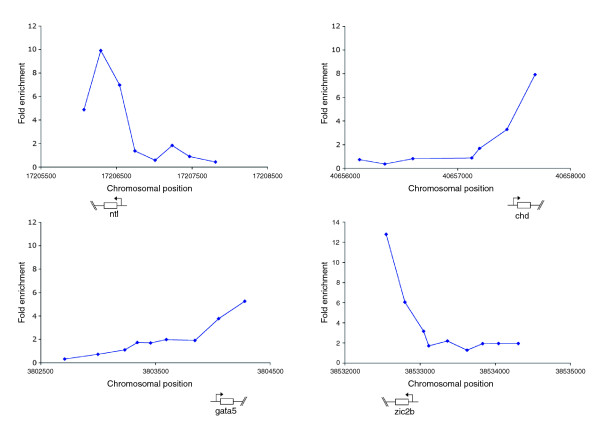
H3K4Me3 is enriched at the 5' end of genes expressed in localized domains of the embryo. Plots show unprocessed ChIP-enrichment ratios for all probes within a genomic region. The chromosomal position (based on Zv4 genome assembly annotation) and the transcription start site and direction of transcription for each gene are shown below each graph.

We find that in many cases genes that are expressed in only a subset of gastrula cells can also be reliably detected by this technique, notwithstanding our false-negative observations above. The gastrula stage embryo is sub-divided into three germ layers and many genes expressed in just one of these germ layers, or even in a subset of cells within a germ layer, were identified as being marked by H3K4Me3 at their 5' ends. For instance, *bon *and *gata5*, which are expressed throughout the endoderm, were identified in this way. In the mesoderm the pan-mesodermal marker *ntl *is identified, while *chd *(expressed in dorsal axial mesoderm) and *sizzled *(expressed in the ventral most mesoderm) were also identified (Figure 5; Additional data file 5). Finally, we identify ectodermal genes, such as *zic2b *and *cyp26a1 *(Figure 6; Additional data file 5).

## Conclusion

In this paper we describe the creation of zebrafish genomic microarrays and show that these can be employed in combination with chromatin immunoprecipitation to detect genes with promoter sequences associated with DNA binding proteins. We have used these arrays to identify genes marked by H3K4Me3 in gastrula stage embryos and shown that these genes include many that are known to be actively transcribed. Preliminary data (unpublished) indicate that these arrays can also be used successfully with ChIP to identify targets of spatially restricted transcription factors in both gastrula and adult zebrafish tissues, such as liver.

The techniques and reagents we report here pave the way for studying genome-wide binding of regulatory factors during vertebrate embryogenesis. This will be important since understanding gene regulation in vertebrate model systems is crucial for understanding human development; in the future this knowledge may allow us more precisely to manipulate embryonic stem cells *in vitro *to form different cell and tissue types. The zebrafish model is an ideal system in which to study transcriptional regulatory networks on a genome-wide scale in developing vertebrates since zebrafish produce hundreds of embryos at each mating and the early embryos develop externally, allowing easy collection and manipulation. In contrast, studies during early embryogenesis in mammalian embryos are technically challenging due to the large numbers of cells currently needed for this technique. Thus, the availability of a genomic platform to study factor binding in zebrafish now enables us to define transcriptional networks in the developing embryo on a genome-wide scale.

## Materials and methods

### Chromatin immunoprecipitation

A detailed protocol for ChIP-chip is given in Additional data file 2. Briefly, for each immunoprecipitation approximately 1,000 mid-late gastrula stage embryos (75% to 85% epiboly) were enzymatically dechorionated and then fixed in 1.85% formaldehyde in 1X embryo medium for 20 minutes at room temperature. For conventional ChIP, approximately 200 embryos were used. Glycine (0.125 M) was added to quench the formaldehyde and the embryos were washed in ice cold 1X PBS and snap frozen on liquid nitrogen or used immediately. Fixed embryos were homogenized in lysis buffer and incubated for 20 minutes on ice. Nuclei were collected by centrifugation, resuspended in nuclei lysis buffer then incubated for 10 minutes before diluting with immunoprecipitation (IP) buffer and sonicating the chromatin sample on an ice bath. Sonication conditions were optimized to give fragments of approximately 300 to 700 bp. The lysate was incubated overnight at 4°C with 100 μl of protein G magnetic Dynabeads (Invitrogen, Carlsbad, CA, USA) that had been prebound to 6 μg of the appropriate antibody. Beads were washed five times with RIPA buffer and once with 1X Tris buffered saline (TBS) at 4°C. Bound complexes were eluted from the beads at 65°C with vortexing in elution buffer. Cross links were reversed for 6 hours at 65°C and the chromatin purified by treatment with RNase A, followed by proteinase K digestion and phenol:chloroform:isoamyl alcohol extraction. Three separate ChIP-chip experiments were carried out on three separate batches of embryos.

### DNA amplification and labeling

For ChIP-chip, purified DNA from anti-histone chromatin immunoprecipitation was blunted using T4 polymerase and ligated to linker. DNA was then amplified using a two-stage PCR amplification protocol. Amplified DNA was labeled and purified using Bioprime Array CGH random prime labeling and purification kit (Invitrogen). Anti-H3 sample was labeled with Cy3, anti-H3K4me3 sample was labeled with Cy5. Labeled DNA for each channel was combined and hybridized to arrays in Agilent hybridization chambers for 40 hours at 40°C. Arrays were then washed and scanned.

### Antibodies

Antibody against Histone H3 (ChIP grade, Abcam 1791, Cambridge, UK) was used to immunoprecipitate input DNA against which DNA immunoprecipitated with anti-H3K4Me3 (ChIP grade, Abcam 8580) was compared. The anti-H3K4Me3 antibody is also reported to recognize weakly di-methylated K4 of Histone H3 [51], a chromatin mark also associated with actively transcribed genes in higher eukaryotes [10,30].

### Embryos

Embryos for each ChIP experiment were collected from crosses of AB strain fish and were collected at 75% to 85% epiboly. Embryos from several different crosses on several different days were pooled for large scale chromatin immunoprecipitation.

### Genomic array design

Microarrays were designed as described below and manufactured by Agilent Technologies [52]. Further information on design can be found at the Whitehead Institute Bioinformatics website [53].

#### Selection of transcription start sites and identification of promoter sequences

We interrogated five databases: Ensembl, VEGA, Refseq, ZGC full length clones and a database provided by Dr Leonard Zon (Harvard Medical School, Boston, USA) in order to assemble an extensive list of zebrafish transcripts. The Zon lab database is a hand-curated database of zebrafish genes that have homologues in other species.

Since extraction of promoters requires accurate identification of the 5' ends of full-length transcripts, we limited ourselves to databases designed to include full-length cDNAs. We chose the above databases rather than EST-based resources such as UniGene, which are much more useful for the design of expression microarrays than promoter microarrays. As a result, the array was designed to include a high-confidence set of promoters, rather than a more inclusive set of promoters that may be prone to errors.

We included all transcripts that appeared in the manually annotated databases (VEGA, Zon) and in the ZGC full length database. We also identified genes present in any two of the five databases and included those not already selected. The transcripts were mapped to the zebrafish genome (Zv4, June 2004) obtained from UCSC Bioinformatics [54] and the TSS for each transcript was determined. Transcripts with TSSs within 500 bp were clustered into a transcriptional unit (TU) and promoter regions were identified relative to the most upstream TSS. This resulted in the identification of 13,413 TUs and corresponding promoter regions. Each promoter region was extracted and masked for repetitive sequence by RepeatMasker [55]. If the promoter region contained a gap, the upstream sequence was also masked. Information on the transcriptional units that were included in the final design can be found at the Whitehead Bioinformatics website [53].

#### Selection of oligonucleotides

We then designed 60-mer oligonucleotide probes representing the region between 1.5 kb upstream and 0.5 kb downstream of the annotated TSS of each transcriptional unit. Although transcription factors and other DNA binding proteins are known to regulate genes from distances of greater than -1.5 kb or +0.5 kb, much information can be gained from regions close to the TSS [50], and the H3K4Me3 mark studied in this paper is found at the most 5' end of a gene, close to the TSS.

Selection of 60-mers for the microarrays was essentially as described in [14] using the Zv4 build of the zebrafish genome and a locally customized version of ArrayOligoSelector [56,57]. We chose 60-mers so that promoter regions contained approximately one probe every 250 bp, with a maximum distance between probes for each promoter region set at 600 bp. In cases where only one probe could be designed for a particular TU, these were not included in the final design. This process yielded 80,839 probes for 11,171 promoter regions.

We also incorporated several sets of control probes, both positive and negative. On each array there are 1,090 probes designed against 'gene desert' regions, which are genomic regions that are unlikely to be bound by transcriptional regulators, and 270 probes designed against *Arabidopsis thaliana *genes, which are not present in the zebrafish genome (by BLAST). In addition, because our main motivation for making these microarrays is to identify mesodermally regulated genes we included seven genes expressed in mesoderm during gastrulation as positive controls (*wnt11*, *flh*, *vent*, *msgn1*, *myod*, *fgf8*, *pcdh8*). Probes designed against these promoters, which flank from 3 to 4 kb around each TSS, are arrayed 2 to 4 times on each slide. Since these genes are expressed at gastrula stages to varying degrees, they also serve as positive controls in this study. Finally, there are 2,256 controls added by Agilent and a variable number of blank spots. These probes were divided between two microarray slides each with 44,290 features.

We refer to these two microarray slides as the 'proximal promoter set'. A proximal promoter set based on these designs as well as an expanded set of 9 slides that contain regions from -9 kb to +3 kb relative to the TSS, are available by contacting Agilent [52] or by downloading the design files from the Whitehead Bioinformatics website [53] for self-manufacture.

### Data analysis

Arrays were scanned using an Axon Instruments Genepix 4000B microarray scanner and Genepix software (Molecular Devices, Sunnyvale, CA, USA) was used to obtain background-subtracted intensity values for each fluorophore for each feature on the array.

#### Data normalization, analysis and identification of bound regions

Analysis was based on [14] with modifications. Data were set-normalized using a collection of 1,360 control probes. A whole chip error model [58] was used to calculate X scores for each spot based on both the absolute value of intensities and background noise in each channel. The X scores are assumed to be normally distributed, which allows for the calculation of a p value for the enrichment ratio seen at each feature. To identify bound probes we initially selected an X score cut-off that would give 5% false positives assuming a normal distribution of non-bound probes on each slide. Any probe that returned an X score ≥2.76 on slide 1 (standard deviation = 1.38) or 2.78 on slide 2 (standard deviation = 1.39) was included in our list of bound probes. We next used an algorithm that incorporates data from neighboring probes to calculate a p value for a group of 3 neighboring probes (probe set p values). Taking neighboring probes into account we required that multiple probes in the probe set provide evidence of binding, so that if the probe set p value was less than or equal to 0.001 the central probe of that set was marked as bound (Additional data file 4).

#### Annotation of bound regions

Each probe was independently mapped back to the zebrafish genome (Zv4) and assigned to a transcriptional unit if it overlapped a promoter, as defined by the region from -1.5 kb to +0.5 kb relative to the corresponding TSS. In some cases a probe was assigned to two promoter regions due to overlapping TUs, either on the same or different strand. Some probes mapped to multiple locations in the genome and these were removed from the final analysis (excluding repeated TUs). In addition, we expect some misannotation of probes due to errors in the genome assembly, which is not yet complete, and errors in identifying transcript sequences from public databases, especially those transcripts that are missing their 5' ends.

#### Creation of meta-gene from regions containing H3K4Me3

The set of 2 kb regions that contained positive enrichment in the H3K4Me3 mark were collected and used to assemble a metagene of the average composite binding *in vivo*. Each region was queried for probes and these were mapped into a set width 2 kb window at the appropriate offsets based on the strand orientation, thus removing strandedness from the calculation. Linear interpolation was used to estimate continuously the fold-enrichment at each base-position within the 2 kb window. This interpolation leaves the 5' and 3' ends of the window somewhat under-represented, and subject to higher variability.

The metagene was then created from this collection of continuous functions by calculating the mean of the values mapped to each position by all the regions enriched by H3K4Me3 ChIP. If the offset proved to correspond to the exact location of a probe within a particular tiled region, the values are directly experimentally measured; alternatively, the value is calculated by linear interpolation of the two nearest flanking probes, as described above.

### Selection of non-expressed and expressed gene lists for estimation of false positive and false negative rates

To estimate the rates of false positives and false negatives we compiled a list of genes that are not expressed at gastrula stages ('non-expressed controls') and genes that are known to be expressed at gastrula stages ('expressed controls'). These control genes were selected from expression information published by Mathavan *et al*. [33] on the zebrafish transcriptome during development, from egg to 48 hours post fertilization. Expressed controls were selected from those that showed peak expression at gastrula stages in the transcriptome data tables [59]. From this list we eliminated genes that were not represented on our arrays. In addition, we selected an additional set of expressed controls that are involved in mesodermal patterning, and thus expressed at gastrula stages and are of particular interest to us, giving a final list of 123 expressed genes. Non-expressed genes were similarly selected from transcriptome analysis data tables and checked for inclusion on our arrays, and included those that were not expressed at any time during development, and those that were not expressed at gastrula stage expression but had high levels of expression in the egg, at 24 hours or at 48 hours. For these non-expressed controls we then searched the ZFIN *in situ *hybridization gene expression database [44] and the Unigene EST ProfileViewer [60] and excluded those genes that had expression reported at gastrula stages in these databases, yielding a final list of 86 non-expressed controls.

The false negative rate was calculated after performing the RT-PCR analysis as follows: since 5/9 (approximately 55%) of the genes on our 'non-expressed' controls list are both called as bound and expressed, we assume approximately 55% of all 15 positive genes are also expressed. This gives approximately 8 genes that are both called as bound and expressed and this number was then removed from the 'non-expressed' control list, leaving 78 'non-expressed' genes. Since approximately 7 of the genes called as bound are not expressed (based on RT-PCR results as described above), this leads to a false positive rate of approximately 9%.

### PCR analysis

During optimization of the ChIP protocol PCR analysis was performed to confirm the enrichment of known positive target promoters (*wnt11 *and *bactin2*) and non-enrichment of a negative target promoter (*rhodopsin*). Primers and cycling conditions are shown in Additional data file 3. Products were run on 1.6% agarose gel and stained using SYBR gold (Invitrogen). Chromatin immunoprecipitation with non-specific antibodies, such as anti-HA (Roche, Basel, Switzerland) or with normal rabbit serum showed no enrichment of any target promoters above background.

### RT-PCR analysis

Total RNA was isolated from embryos and cDNA prepared by standard protocols. The cDNA from 75% to 85% epiboly embryos was used as a template in semi-quantitative PCR. For a control sample, cDNA from unfertilized eggs, 75% to 85% epiboly embryos and 24 hours post-fertilization embryos was mixed in equal quantities (each gene tested has reported expression at 24 hours post-fertilization or in eggs) and serially diluted to test the number of cycles required to capture the reaction in its linear range. For the analysis shown in Figure 3, 20 ng of 75% epiboly cDNA and an equivalent reaction volume of minus reverse transcription (-RT) reaction was used; the control sample was 60 ng of mixed stage cDNA (20 ng of each stage). Primers and cycling conditions are shown in Additional data file 3.

### Data availability

Complete, unprocessed data have been deposited into the public database Gene Expression Omnibus [61] with the accession number GSE4863. Additional analysis containing p values and ratios for bound regions can be found on the Smith lab worldwide web site [62].

## Additional data files

The following additional data are available with the online version of this paper. Additional data file 1 is a figure showing an example of PCR analysis on ChIPed DNA. Additional data file 2 describes the zebrafish ChIP-Chip protocol, giving detailed information of the experimental procedure used. Additional data file 3 lists the primers used in RT-PCR validation; the sequences of primers used in reverse transcription-PCR analysis and cycling conditions for each primer pair are given. Additional data file 4 lists probes showing enrichment in ChIP-chip analysis. Each probe is given a chromosome and chromosomal position based on Zv4 zebrafish genome assembly. Additional data file 5 lists genes and transcripts associated with a genomic region or probe that is enriched in ChIP-chip analysis. Additional data file 6 lists genes that are reported not to be expressed at gastrula stages and indicate if the gene is bound (probes at the 5' end of the gene are enriched in ChIP-chip analysis), or not bound (probes show no significant enrichment). The table also shows the results of RT-PCR analysis on selected genes. Additional data file 7 lists genes that are reported to be expressed at the gastrula stage and indicate if the gene is bound (probes at the 5' end of the gene are enriched in ChIP-chip analysis), or not bound (probes show no significant enrichment).

## Supplementary Material

Additional data file 1 Anti-H3 and anti-H3K4Me3 antibodies were use to ChIP DNA from gastrula stage embryos. PCR was performed on this DNA and on whole cell extract DNA that was not immunoprecipitated with primers designed against the promoter region of *rhodopsin*, *bactin2 *and *wnt11*. As expected, *rhodopsin*, which is not expressed at gastrula stages, was not enriched over anti-H3, where as *bactin2*, which is expressed ubiquitously, and wnt11, which is a mesodermally expressed gene, are both enriched over H3. Click here for file

Additional data file 2 The zebrafish ChIP-Chip protocol, giving detailed information of the experimental procedure used Click here for file

Additional data file 3 Sequences of primers used in RT-PCR analysis and cycling conditions for each primer pair are given Click here for file

Additional data file 4 Probes showing enrichment in ChIP-chip analysis. Each probe is given a chromosome and chromosomal position based on Zv4 zebrafish genome assembly Click here for file

Additional data file 5 Genes and transcripts associated with a genomic region or probe that is enriched in ChIP-chip analysis Click here for file

Additional data file 6 Genes that are reported not to be expressed at gastrula stages and indicate if the gene is bound (probes at the 5' end of the gene are enriched in ChIP-chip analysis), or not bound (probes show no significant enrichment). The table also shows the results of RT-PCR analysis on selected genes Click here for file

Additional data file 7 genes that are reported to be expressed at the gastrula stage and indicate if the gene is bound (probes at the 5' end of the gene are enriched in ChIP-chip analysis), or not bound (probes show no significant enrichment) Click here for file
