# Supplementary material for: Zebrafish promoter microarrays identify actively transcribed embryonic genes
Source: Genome Biol. 2006 Aug 4;7(8):R71. doi: 10.1186/gb-2006-7-8-r71 (PMC1779600; doi:10.1186/gb-2006-7-8-r71)
Supplement: Additional data file 2 — The zebrafish ChIP-Chip protocol, giving detailed information of the experimental procedure used [file gb-2006-7-8-r71-S2.pdf]

Zebrafish ChIP-array protocol: **version 1 August 5th 2005**

PROCEDURE

I. Fixation of embryos
1. Dechorionate embryos (Pronase treatment)
2. Fix embryos, 15 mins in 1.85% formaldehyde in Embryo medium, RT, rotating.
3. Add 1/20th volume of 2.5M glycine, to quench formaldehyde. 5mins, RT, rotating.
4. Rinse embryos 3 x in ice cold 1X PBS. (At this point embryos can be snap frozen for future use).

II. Preblock and binding of antibody to magnetic beads (@4°C preferred)
1. Wash 100-200 µL Dynal Protein A or G magnetic beads (per reaction) in 1 ml fresh BSA/PBS.
1. Collect the beads by spinning at 3000 rpm for 3 minutes.
1. wash beads in 1.5 ml BSA/PBS 2 times, collect the beads with the magnet.
1. Add 6-10 µg of Ab + 250µL of PBS/BSA.
1. Incubate 4 hr to O/N on a rotating platform at 4°C.
1. Wash beads 3 times in 1.5 ml PBS/BSA.
1. Resuspend in 100µL PBS/BSA.

III. Preparation of chromatin
1. Lyse embryos in 2ml cell lysis buffer (histone ChIP= ~1000 embryos per condition). Pipette up and down or use pestle to homogenize, leave on ice for 15mins, pipette up and down again to ensure embryos are completely lysed.
2. Spin at 3,500rpm, 5 mins, 4˚C to pellet nuclei.
3. Resuspend pelleted nuclei in 1ml nuclei lysis buffer.
4. Pipette up and down to disrupt nuclei. Lay tube on ice (if too cold SDS will precipitate) and incubate for 10 mins.
5. Add 2 volumes of IP dilution buffer.
6. Sonicate the suspension in 15ml conical tube. Sonicate 6-10 times for 10 sec, allowing the suspension to cool on ice for 1 min between pulses. Sonication conditions should be determined empirically for individual sonicator (see solutions and additional protocols).
7. Add 240ul 10% Triton to 3ml of chromatin.
8. Transfer into 1.5ml tubes and spin at 14,000 rpm, 4°, 10 mins. Transfer the supernatant to a new 15ml tube.
9. Save 50 µl of cell lysate from each sample as input control. Store at -20°C.

IV. Chromatin Immunoprecipitation
1. Add 100 µl Ab prebound Dynal magnetic beads from step III.
2. Rock 4°C O/N

V. Washing, eluting, and reverse cross-linking
1. Transfer to centrifuge tubes, continue working in the cold room until step 6.
1. Use the magnetic stand to precipitate the beads.
1. Save supernatant from step 2. – use in Western analysis to check depletion of protein (see solutions and additional protocols).
1. Wash 4-8 times with 1 ml RIPA wash buffer
1. Wash once with 1 ml 1X TBS.

2. Spin 3k for 2-3 min and aspirate any residual TBS.
3. Add 210 µl of elution buffer.
4. Elute DNA-protein complexes from beads at 65°C for 10-15 min with brief vortexing every 2 min.
5. Spin down beads 14k for 1 min.
6. Remove 200µl of supernatant (or leave beads in while reversing cross-links).
7. Reverse x-link 6 hours - O/N, 65°C.

12. Thaw input from step III(9), add 3 vol of elution buffer and reverse x-link 6 hours - O/N, 65°C.

VI. Clean up

1. Add 1 vol (200µl) of TE to IP and input fraction.
2. Add RNase A so final is 0.2µg/µl (8µl of 10mg/ml per 400µl rxn). Incubate 37°C 1-2hr.
3. Add proteinase K (Invitrogen) so final conc. is 0.2µg/µL (4µl of 20mg/ml per 400µl rxn). Incubate 55°C 1-2hr.
4. Extract once with 1 vol phenol:chl:IA using phaselock tubes
5. add 20 µg (1 µl) of glycogen.
6. Add 5M NaCl so final conc. is 0.2M (16 µl/400 µl rxn).
7. Precipitate DNA with a 10 min 14K spin, and wash pellet with 500 µl 75% EtOH.
8. Dry and resuspend pellets in 70 µl 10mM Tris HCl pH 8. Save up to 15 µl of IP sample for checkpoints (see solutions and additional protocols). Normalize the wce fraction to 100 ng/µl using the Nanodrop.

VII. T4 Fill-in and blunt-end ligation

A. T4 DNA polymerase fill in

1. To 55 µl of IP sample, and 200 ng (=2 µl) of the normalized wce diluted to 55 µl add:
   - 11 µl 10X T4 DNA pol buffer
   - 0.5 µl BSA (NEB 10mg/ml)
   - 1 µl 10mM dNTP mix
   - 0.2 µl T4 DNA pol (NEB 3U/µl)
   - up to 110 µl H_2O
2. Transfer sample to PCR tube and incubate 12°C 20 min.
3. Add 11.5 µl 3 M NaAc and 0.5 µl (=10 µg) glycogen.
4. Extract 1x with 120 µl phenol:chl:IA.
5. Precipitate with 250 µl EtOH.
6. Spin and wash with 500 µl 75% EtOH.
7. Dry pellet and resuspend in 25 µl H_2O.

B. Blunt-end ligation

1. Make at 4°C, 25 µl ligase mix per reaction:
   - 7.8 µl H_2O
   - 10 µl 5X ligase buffer (Invitrogen)
   - 6.7 µl annealed linkers (*thaw at 4°C*)
   - 0.5 µl T4 DNA ligase
2. add mix to 25 µl of sample.
3. incubate 4°C or 12°C O/N, cover with foil.
4. Next day add 6µl of 3M NaOAc and 130 µl EtOH.
5. Spin and wash with 500 µl 75% EtOH.
6. Dry and resuspend pellet in 40 µl PCR reaction mix below.

VIII. Ligation mediated PCR

1. for PCR: use pellets from blunt ligation.
2. Make PCR mix per rxn:
   33.5 µl H_2O
   4 µl 10X Thermopol buffer (NEB)
   1.25 µl 10 mM dNTP mix
   1.25 µl 40 µM oligo oJW102
3. Dissolve pellet in mix above and start PCR program: 55 °C _ 4'; 72°C _ 3'; 95°C _ 2'; 95°C _ 30''; 60°C _ 30''; 72°C _ 1'; goto step 4 14 times; 72°C _ 1'; 4°C _ hold.
4. During step 1 (55 °C _ 4') add Taq mix:
   8 µl H_2O
   1 µl 10X ThermoPol buffer (NEB)
   0.5 µl Taq (5U/µl)(Applied Biosystems)
5. For expansion: take 50ul rxn and add 450µl 10mM Tris pH8. Take out 5ul and add 45µl PCR mix (below). Leaves 495µl for further expansion (e.g. onto 9 array set).
6. PCR mix per rxn:
   37.25µl H_2O
   5µl ThermoPol buffer
   1.25µl 10mM dNTPs
   1.25µl 40µM oligo oJW102
   0.25µl Taq (5U/µl)
   5µl template
7. Place in thermocycler and start PCR program: 95°C _ 2'; 95°C _ 30''; 60°C _ 30''; 72°C _ 1'; goto step 4 24 times; 72°C _ 5'; 4°C _ hold.
8. Take out 3ul to run on gel (see solution as additional protocols at end)
9. Add 25µl of 7.5M NH_4OAc and 225µl EtOH. Spin and wash pellet with 500 µl 75% EtOH. Redissolve in 50 µl H2O.
10. Normalize [DNA] to 100 ng/µl.

IX. Cy3-Cy5 Random Primer labeling

1. For each slide use 1 µg of DNA from normalized IP and 1 µg input LM-PCR product above (=10µl of LM-PCR product).
2. Add 30 µl water to each 10µl DNA sample.
3. Add 35 µl of 2.5X random primer solution (Invitrogen Bioprime labeling kit).
4. Boil 5 min in heatblock (Use cap lock). Place tubes in icewater. Incubate 5 min.
5. Add 1.8µl water and 8.2 µl 10X low T dNTP mix (1.2 mM dATP, dCTP, dGTP each and 0.6mM dTTP).
6. Add 1.5 µl of cy5-dUTP to IP tube and 1.5 µl cy3-dUTP to input tube (by convention).
7. Add 1.5 µl of high concentration Exo-Klenow (40U/µl, Bioprime kit) to each tube..
8. Incubate RT (20 °C) 16 hrs (keep samples in dark as much as possible from here on. e.g. put in drawer overnight). Or incubate at 37˚C for a few hours – but gives lower signal.
9. Cleanup labeling rxn with Bioprime CGH columns: to 100µl reaction add 500µl Buffer A. Add to column, spin 1min, 13,000rpm. Wash with 600µl Buffer B, then 200µl. Elute with 50µl water, stand 1min, spin 1min. (Nanodrop to determine conc of DNA).
10. Precipitate rxn with 2.5 volumes EtOH, 0.5 volumes 7.5M Ammonium Acetate (not sodium acetate). Wash in 75% EtOH.
11. Dry samples at 37°C (>10 min)

12. Dissolve colored pellet in 50 µl H_2O and nanodrop again on microarray program (measure conc. of DNA and Cy5 or Cy-3 (pM/µl))

X. Chip hybridization
Probe preparation.

1. For each array, combine 5µg Cy3 with 5µg Cy5 sample.
2. Make following 2 mixes:
    1. 1µl Herring Sperm DNA 10µg/µl
       5ul tRNA 10µg/µl
       40µl 1X Agilent control targets (cat no: 5185-5976)
    2. 50µl 500mM Na-MES pH6.9
       50µl 5M NaCl
       6µl 500mM EDTA
       50µl 5% Sarcosine (Fluka)
       150µl Formamide
       41.5µl water
3. Combine mixes with Cy3/5 DNA sample (comes to 500µl. Keep at RT, under foil).
4. Set hyb oven to 40˚C. Have a heatblock at 100˚C and another at 40˚C.
5. Boil samples for 3 mins, cool to 40˚C (1-15mins). Spin to collect sample.
6. Meanwhile set up hyb chamber: put clean slide gasket, with gasket uppermost, in slide holder.
7. Place 470µl of hyb solution in middle of gasket slide. Place array slide onto gasket slide, “Agilent” facing down, barcode facing up.
8. Screw slide holder on top, so liquid is 2/3rds to 3/4trs up chamber. Rotate slide once to check for bubbles. Knock out bubbles if necessary. Next day, check for bubbles and knock out if needed.
9. Incubate slides with rotation at 40˚C for 40-48hrs (less gives less signal, longer signal also starts to deteriorate).

XI. Slide washing

1. Rinse all basins etc in distilled water to remove dust, also rinse gloves. Do not dry.
2. Fill bowl and one 250ml glass wash jar with buffer 1. Fill 2 x 250ml glass wash dishes with Buffer 2.
3. Break slides open by taking slide sandwich out of holder with flat forceps, place into bowl at angle and use forceps to split sandwich apart. Hold onto array slide and swish back and forward twice.
4. Transfer slide into rack in wash dish containing Buffer 1. Shake 65 rpm for 5 min, RT.
5. Transfer rack with slide(s) into glass dish containing Buffer 2 to rinse.
5. Transfer slides into second glass dish containing Buffer 2. Shake 65rpm, 5 min.
6. Slowly pull out rack with slides from Buffer 2. Blot side of slides to remove excess buffer.
7. Transfer into 50ml Coplin jar of Agilent Stabilization and Drying solution (cat no: 5185-5979).
8. Incubate slides for 30 secs, then pull put slowly to count of ~15. Slides should be dry.
9. Scan.

Solutions and additional protocols

I. Fixation of embryos

1.85% Formaldehyde Solution in EM
500µl 37% Formaldehyde
9.5 ml Embryo Medium

2.5 M glycine
3.75g in 50ml H_2O
filter and store at RT.
Do not adjust pH

II. Preblock and binding of antibody to magnetic beads

0.5% BSA in 1X PBS
50mls 1X PBS
0.25g BSA
Store at 4˚C, good for a week.

III. Preparation of chromatin

Cell Lysis Buffer (use @4°C)
Final conc.
10mM Tris-HCl (pH7.5)
10mM NaCl
0.5% NP-40

Nuclei Lysis Buffer
Final conc.
50mM Tris-HCl (pH7.5)
10mM EDTA
1% SDS

IP Dilution Buffer (use @4°C)
Final conc.
16.7mM Tris-HCl (pH7.5)
167mM NaCl
1.2mM EDTA
0.01% SDS

Note: use Tris pH7.5 for protein G and pH8 for protein A
Note: Add protease inhibitors (Complete tablet, Roche) to all lysis buffers before use.
Note: If pre-clearing lysate, do so before section III, step 9.

Testing sonication conditions required. To check sonication conditions take a 50µl sample from sonicated chromatin and reverse crosslinks at 65˚C as described above. Clean DNA, precipitate and resuspend in 10µl. Run 5µl of this sample on a 1.6% agarose gel and check spread. Aiming for DNA to be ~300-700bp.

V. Washing, eluting, and reverse cross-linking

Wash buffer (RIPA buffer)
final conc.
50 mM Hepes (pH 7.6)
1 mM EDTA
0.7% DOC
1% IGEPAL
0.5 M LiCl

Elution buffer:
final conc.
50mM Tris pH8
10mM EDTA
1% SDS

Western analysis
Use unbound supernatant from IP to check for depletion of antibody.
OR
-Use a portion of elution to reverse x-link
-Check for precipitated protein by Western analysis after crosslink reversal

VI. Clean up

Fragment Size Verification
After sonication, the spread of size fragments can be checked by running 1 µl of wce DNA on a 1-2% agarose gel. Spread should be concentrated around 500bp

Gene specific PCR
Check for IP enrichment specific for transcription factor by performing gene specific PCR with 2-3 µl of IP sample and a dilution series of input sample. Enrichment should be at least 2 fold over a non specific gene.

VII. Blunt-end ligation

Oligos for blunt end ligation:
oJW102:
5'-GCGGTGACCCGGGAGATCTGAATTC
oJW103:
5'-GAATTCAGATC

Final buffer concentrations for annealing:
50 mM Tris pH 7.5
50 mM NaCl
17.5 µM each oligo
After mixing, boil 5 minutes, and anneal slowly from a 100°C heat block to 25°C, then cool to 4°C overnight, aliquot, and freeze. Do not re-use once thawed.

VIII. Ligation mediated PCR

Product Size and Amount Verification
Remove 3 µl of LM-PCR product and run it out on 2% agarose gel to check integrity.

XI. Slide washing

Wash buffer 1
Make fresh
600ml 20x SSPE
2ml 5% Sarcosine
make up to 2 litres with water. Filter.

Wash buffer 2
Make fresh
6ml 20x SSPE
make up to 2 litres with water. Filter.
